# Advances in the Knowledge about Kidney Decellularization and Repopulation

**DOI:** 10.3389/fbioe.2017.00034

**Published:** 2017-06-01

**Authors:** Afrânio Côgo Destefani, Gabriela Modenesi Sirtoli, Breno Valentim Nogueira

**Affiliations:** ^1^Tissue Engineering Core—LUCCAR, Morphology, Federal University of Espírito Santo (UFES), Vitória, Brazil; ^2^Health Sciences Center, Federal University of Espírito Santo (UFES), Vitória, Brazil; ^3^Health Sciences Center, Postgraduate Program in Biotechnology/RENORBIO, Vitória, Brazil

**Keywords:** kidney transplantation, bioengineering, decellularization, regenerative medicine, stem cell

## Abstract

End-stage renal disease (ESRD) is characterized by the progressive deterioration of renal function that may compromise different tissues and organs. The major treatment indicated for patients with ESRD is kidney transplantation. However, the shortage of available organs, as well as the high rate of organ rejection, supports the need for new therapies. Thus, the implementation of tissue bioengineering to organ regeneration has emerged as an alternative to traditional organ transplantation. Decellularization of organs with chemical, physical, and/or biological agents generates natural scaffolds, which can serve as basis for tissue reconstruction. The recellularization of these scaffolds with different cell sources, such as stem cells or adult differentiated cells, can provide an organ with functionality and no immune response after *in vivo* transplantation on the host. Several studies have focused on improving these techniques, but until now, there is no optimal decellularization method for the kidney available yet. Herein, an overview of the current literature for kidney decellularization and whole-organ recellularization is presented, addressing the pros and cons of the actual techniques already developed, the methods adopted to evaluate the efficacy of the procedures, and the challenges to be overcome in order to achieve an optimal protocol.

## Introduction

Chronic kidney disease (CKD) and its complications are of paramount importance in the context of public health (Lai et al., 2013), reaching approximately 8–16% of the adult population worldwide (Jha et al., 2013; United States Renal Data System, 2015). Its overall prevalence progresses at an alarming rate and is correlated with the dramatic increase in obesity in the last 30 years, leading to Type II diabetes, metabolic syndrome, and renal failure—also known as end-stage renal disease (ESRD) (Nguyen and El-Serag, 2010; Yokote et al., 2012). In the USA, it is estimated that one in every ten Americans, approximately 23 million people aged 20 years or older, has some form of CKD [Centers for Disease Control and Prevention (CDC), 2014], and this number is expected to rise given that the elderly population is also growing (Murray et al., 2013).

Although the incidence of CKD is rising, the available therapeutic options remain limited (Levey and Coresh, 2012). Current therapeutic options for renal replacement are limited to peritoneal dialysis, hemodialysis, or kidney transplantation (KT). Renal transplantation is the ideal treatment for patients with ESRD: it improves long-term survival (Wolfe et al., 1999), leads to a better quality of life (Dew et al., 1997; Anil Kumar and Mattoo, 2015), and is effective in terms of cost compared with long-term dialysis (Hutton, 1999). The success of renal transplantation depends on the donor type, age, sex, race, and primary cause of ESRD. In general, the 1-year graft survival and patient survival advantage experienced by living donor transplant recipients persist at 5 and 10 years post-transplant (USRDS, 2016). According to the United States Renal Data System, in 2013, the rate of 5-year graft survival was 73% for recipients of deceased donor transplants, while it was 85% for living donor transplant recipients (United States Renal Data System, 2015). However, KT is often associated with chronic dysfunction linked to immune rejection (National Kidney and Urologic Diseases Information Clearinghouse, 2012). In addition, the significant increase in the use of living kidney donors and cadavers is not compatible with the availability of organs; in the last years, there were more than 100,000 patients in the USA waiting for a transplant with an average of more than 3.6 years (Matas et al., 2015; United States Renal Data System, 2015).

## Alternative Management of Kidney Disease

For patients with ESRD, KT is closely associated with an increase in life expectancy, improving the quality of life, and is a cost-effective therapy (Wolfe et al., 1999; Johnson et al., 2000; Ojo et al., 2001; Merion et al., 2005; Tonelli et al., 2011; Rana et al., 2015). To emphasize further this growing disparity between the supply and demand for organs, the total number of KTs performed annually in the United States has remained static over the last decade (Valapour et al., 2014). Furthermore, there are an increasing number of elderly donors and recipients for KT (Wolfe et al., 2010; Abecassis et al., 2012). US initiatives, for example, have resulted in an increase in non-conventional sources of donors, such as expanded criteria donors (ECD) (Port et al., 2002; Stratta et al., 2006; Pascual et al., 2008; Klein et al., 2010), donors after cardiac death donation (DCD) (Howard et al., 2005; Abt et al., 2006; Farney et al., 2011), standard criteria donors (SCD) with warm ischemia times or prolonged cold (CIT) (Roodnat et al., 2003; Kayler et al., 2011; Kim et al., 2013; Debout et al., 2015; Xia et al., 2015), acute kidney injury (AKI) donors (Anil Kumar et al., 2006; Kayler et al., 2009; Farney et al., 2013; Hall et al., 2015; Heilman et al., 2015; Xia et al., 2015), double-kidney transplantation (DKT) and donors at the extremes of age (Johnson et al., 1996; Cruzado et al., 2007; De Serres et al., 2010; Fernández-Lorente et al., 2012). Recently, it was demonstrated that desensitization of the patient and subsequent transplantation with a kidney from an incompatible live donor increase the patient survival rate compared with those who remain on the waiting list for transplantation (Orandi et al., 2016). However, all of these alternatives have the drawback of requiring donors.

Several studies have shown that regenerative therapy using mature adult or stem cells may have success. They have demonstrated that postnatal mammalian kidneys may be partially repaired after resection, and this regeneration is mainly due to the proliferation of mature surviving cells or stem cells present in the kidney, such as glomerular parietal epithelial cells (a type of renal progenitor cell) (Vogetseder et al., 2008; Swetha et al., 2011; Wen et al., 2012; Franquesa et al., 2013). After stress, these cells migrate to the damaged area and proliferate and then differentiate into new somatic cells (Hanna et al., 2010; Yamanaka and Blau, 2010; Wen et al., 2012). These results suggest that regenerative solutions offer great potential for the treatment of ESRD. However, achieving a clinically significant regeneration level has proven to be extremely difficult due to the complexity of this organ (Yu et al., 2014).

Another alternative to the shortage of organs would be xenotransplantation using animal organs (Yokoo and Kawamura, 2009; Wang et al., 2014), through a strategy that combines organ decellularization and the insertion of patient’s cells to avoid the risk of adverse immunological reactions (Barakat et al., 2012). The use of pig kidneys for tissue engineering is an attractive approach, mainly because the size of porcine organs is similar to that of humans, and pig scaffolds may allow better adhesion, survival, and maintenance of human cells than scaffolds obtained from dogs or monkeys (Nakayama et al., 2010; Song and Ott, 2011; Faulk et al., 2014a,b; Habka et al., 2015; Moini et al., 2015; Rana et al., 2017; Lih et al., 2016; McKee and Wingert, 2016). The idea of building a functional kidney using specific stem cells from the patient supports a tangible alternative. In the last 2 years, there has been substantial progress toward this goal (Uzarski et al., 2014). In theory, this approach could increase the range of options for KT, enabling the improvement of more patients and potentially minimizing or eliminating the need for long-term immunosuppressive therapy and reducing the waiting time for treatment.

## Tissue Engineering

The increasing number of patients facing end-stage diseases, the shortage of organ donors, and challenges that surround allogenic transplants drive the evolution of tissue engineering and regenerative medicine (Caldas et al., 2011). Tissue engineering is a promising alternative to organ transplantation, with a huge potential in tissue regeneration and repair processes, providing solutions to the conditions in which native tissue is compromised (Lu et al., 2011; He and Callanan, 2013; Teodori et al., 2014). Recent advances in the use of stem cells and regenerative medicine have offered new hope for the treatment of kidney disease, especially ESRD (Krause and Cantley, 2005; Ross et al., 2009; Li et al., 2010).

To reconstruct a new tissue, a triad of components is needed: cells, biomaterials to be used as supports or scaffolds, and growth factors (Ahn et al., 2007; Narayanan et al., 2009; Ng et al., 2011). Biomaterials play a central role because most mammalian cell types are dependent on anchorage (Hollister, 2005; O’Brien, 2011; Cheng et al., 2013; Guan et al., 2015a), and one of the crucial challenges of this area is the selection of the ideal scaffold for cell attachment to ensure its functional integrity. Using a decellularized organ composed of extracellular matrix (ECM), regenerative medicine proposes new horizons (Gilbert et al., 2006; Crapo et al., 2011).

Biological scaffolds consisting of ECM are commonly used for various reconstructive surgical applications, and their use in regenerative medicine for tissue and organ replacement has increased in the last decade. ECM common sources include the dermis, pericardium, small intestine, sub-mucosa, and bladder (Badylak, 2002; Badylak et al., 2009; Rose et al., 2009; Orlando et al., 2011a; Yagi et al., 2013). It is possible to obtain ECM scaffolds from different animals, which can later be recolonized with human cells and then used for transplantation in humans (Cooper et al., 2002).

The ECM is a three-dimensional network that forms a milieu surrounding cells that reciprocally influences cellular function to modulate diverse fundamental aspects of cell biology (Hynes, 2009) such as support for organs and tissues, for cell layers in the form of basement membranes, and for individual cells as substrates for cell motility (Hynes, 2009). Two main classes of extracellular macromolecules make up the matrix (Lai et al., 2013) polysaccharide chains of the class called glycosaminoglycans (GAGs)—a gel-like polysaccharide ground substance, which are usually found covalently linked to protein in the form of proteoglycans, and (United States Renal Data System, 2015) fibrous proteins, including collagen, elastin, and several other molecules (laminins, fibronectins, tenascins) (Brown et al., 2006), which have both structural and adhesive functions (Alberts et al., 2002). GAGs are highly negatively charged and they bind positively charged ions and trap water molecules to form hydrated gels, thereby providing mechanical support to the ECM (Cooper, 2000). In addition, this structure allows nutrition, innervation, cell signaling, and tissue regeneration (Bosman and Stamenkovic, 2003; Naranjo et al., 2009; Ramage, 2011; Keane et al., 2012; Reticker-Flynn et al., 2012). In the kidney cortex, the ECM is present in different anatomical areas, with different functions depending on their molecular components. The ECM present in the interstitial medulla is physiologically more prominent than cortical interstitial ECM and quantitatively greater from the exterior to the interior of the medulla/papilla. The hilar region–renal pelvis (e.g., sub-urothelial basement membrane) and renal capsule also comprise the ECM. Renal interstitial ECM normally consists of collagen type I, III, V, VI, VII, and XV; sulfated- and non-sulfated GAGs; and polysaccharides (Genovese et al., 2014). Specifically, regarding the composition of the ECM of the glomerular basement membrane, the major compounds are laminin, type IV collagen, heparan sulfate proteoglycan (including agrin), and nidogen (Suh and Miner, 2013).

It is possible that scaffolds composed of ECM that integrate the basic structure of the organ that may be used to promote kidney regeneration. In the last decade, numerous studies have demonstrated that constructed ECM scaffolds can support the growth and differentiation of multiple cell types (Reing et al., 2010; Lu et al., 2011; Moroni and Mirabella, 2014). For success clinically, the ECM must be decellularized (Freytes et al., 2008; Choi et al., 2012). It has been shown that decellularized scaffolds may maintain the tri-dimensional composition and biological activity of the ECM; this enables it to be a useful material for cell adhesion, differentiation, and proliferation (Barkan et al., 2010; Vorotnikova et al., 2010; Tien and Nelson, 2014). The advantage of using acellular scaffolds of organs is the possibility not only of using the ECM as a structural scaffold but also allowing the spread of signals that can be exchanged with the cells that adhere to induce migration and differentiation (Gilbert et al., 2007; Gong et al., 2008). The ECM also plays an important role in the maintenance of an adequate network of vessels and growth factors, which allows the appropriate structure of the organ when rebuilt (Ross et al., 2012). These characteristics are indispensable in complex tissues with different cellular components.

Because of the high complexity of the anatomical structure and composition of kidney, renal ECM scaffold has been proposed as a promisor strategy for the reconstruction of a viable kidney. However, obtaining a viable kidney ECM is extremely difficult because of its complex composition, including amino acids, collagens, glycoproteins, GAGs, and microstructures required for filtration (Badylak, 2004; Gagliardini et al., 2010; Mecham, 2001). The appropriate process for the decellularization of tissues/organs must preserve the essential components of the ECM (Song and Ott, 2011). By contrast, synthetic organic scaffolds are inappropriate in terms of complexity. Regarding highly artificial efficient membranes and cell-based bioreactors, there have been successful attempts concerning the miniaturization of the existing bioartificial kidney using various silicon and related thin-film material substrates commonly used in the construction of microelectromechanical systems (MEMS), as well as novel silicon nanopore membranes (SNMs) (Fissell et al., 2006; Fissell and Roy, 2009). The final developmental step based on prior success of the renal tubule cell assist device (RAD) in acute disorders and wearable bioartificial kidney (WEBAK) in preclinical models is the current approach to design, fabricate, and test in preclinical models a functional fully implantable bioartificial kidney (IBAK) (Roy et al., 2011). However, there are still substantial technical challenges regarding the safety and operation of the device, as well as its effectiveness, which need to be overcome (Kooman et al., 2015).

The implantation of renal ECM scaffolds has shown benefit in the promotion of angiogenesis, recruitment of circulating progenitor cells, minimization of immunogenic reactions, and reduction of disease transmission risks (Vavken et al., 2009). Thus, decellularization of an entire organ arises as a novel approach to the generation of a natural 3D architecture.

## Methods and Processes Applied for Organ Decellularization

Decellularization is a process that may involve a blend of chemical, physical, and enzymatic treatments (Bolland et al., 2007; Badylak et al., 2009; Lu et al., 2011; He and Callanan, 2013). More specifically, it may employ detergents, salts, enzymes, and/or physical agents for the removal of tissue and organ cells. There are many existing methods for different applications (Gilbert et al., 2006; Badylak et al., 2011; Gilbert, 2012; Ofenbauer et al., 2015). In general, the process removes the cells present in the tissue, preserving partly the proteins of the ECM, such as collagen, with significantly reduced immunogenic potential (Mirmalek-Sani et al., 2013; Caralt et al., 2015). Thus, the ECM can be repopulated with cells extracted from the patient, creating a new organ with minimal probability of being rejected (Hammerman, 2002; Badylak and Gilbert, 2008).

Currently, the most efficient and robust method for decellularization is the use of chemical infusion. The chemical reagents, particularly detergents or acids, remove native cells by disrupting cell membranes and isolating the cellular components of the ECM (Gilbert et al., 2006). For example, peracetic acid (PAA) is commonly used because it removes nucleic acids with minimal effect on the ECM structure (Gilbert et al., 2008; Gao et al., 2015). Triton X-100, a non-ionic detergent, is the most efficient for the removal of cellular debris from tissues such as heart valves (Liao et al., 2008). Sodium dodecyl sulfate (SDS) is reported to be more effective than Triton X-100 to eliminate cells in the medullary regions of dense organs such as the kidney (Nakayama et al., 2010), and sodium deoxycholate (NaDOC), an anionic detergent, can completely remove the cytoplasmic proteins in dense tissues (Wang et al., 2014).

Decellularized kidneys using Triton X-100 retained growth factors and ECM components, although the cells were not properly removed. On the other hand, decellularization with SDS could sufficiently remove cells while preserving the ECM (Matthiesen et al., 2007; Tanemoto et al., 2008; Nakayama et al., 2010, 2011, 2013; Ross et al., 2012; Sullivan et al., 2012; Caralt et al., 2015). Choi et al. (2015), however, obtained opposite results regarding the comparison between the effects of SDS and Triton X-100 on porcine kidneys. They observed that a richer ECM protein and growth factor content were present in the Triton-treated scaffold than in the SDS-treated scaffold; additionally, cell viability, adherence, and proliferation were higher with the Triton-treated scaffold than with the SDS-treated scaffold (Choi et al., 2015). Furthermore, other researchers have demonstrated that the combination of the two agents produces better results (Sugawara et al., 2011). In view of these divergences, there is still a need to identify more suitable decellularizing agents to improve the quality of the obtained scaffolds (Table 1) (Ross et al., 2009, 2012; Peloso et al., 2013; Song et al., 2013).

**Table 1:** **An overview of the kidney decellularization literature**.

Source kidney	Methods						Overall time	Results	Reference
	Before perfusion	Decellularization solutions	Perfusion pressure	Perfusion flow rate	After perfusion	Threshold			
Goat	Heparinization	0.1% SDS; gradient of SDS (0.5%, 1.0%); 0.1%TritonX-100; 5 mM calcium chloride and magnesium sulfate	100 mmHg at 37°C	Not reported	0.0025% DNase; dH_2_O + 1× PBS containing 10,000 U/mL penicillin G, 10 mg/mL streptomycin and 25 μg/mL amphotericin B at 1 mL/min constant perfusion	Time	5–6 days	Preserved the structure and composition of renal ECM and vascular structures within the scaffold No evidence of residual cellular components was found	Vishwakarma et al. (2014)

Human	dH_2_O at a rate of approximately 12 mL/min for 12 h	0.5% SDS for 48 h	Not reported	12 mL/min	PBS 5 days at a flow rate of 6 mL/min	Not reported	7 days	SDS-based decellularization protocol completely cleared the cellular compartment in these kidneys, while the innate ECM framework retained its architecture and biochemical properties	Orlando et al. (2013)

Human	Placed on ice until decellularization PBS at the rate of 12 mL/min for 12 h	0.5% SDS	Not reported	12 mL/min	DNase for 6 h at a flow rate of 6 mL/h and then with PBS at the same flow rate for 5 days	Not reported	7–8 days	Discarded human kidneys are a suitable source of renal scaffolds because they maintain a well-preserved structure and function of the vasculature, as well as grow factors that are fundamental to achieve a satisfying recellularization of the scaffold *in vivo* due to their angiogenic properties.	Peloso et al. (2015)

Mouse	Physical decellularization: samples were washed in normal saline and stored for 1 week at −4°C. Chemical decellularization.	Nitrogen for 2 min. 1% SDS	Not reported.	Not reported.	PBS at 37°C PBS (24 h)	Time.	1–2 days.	Complete removal of cells and nuclei.	Rafighdoust et al. (2015)

Mouse	Vessels were cannulated and attached to a peristaltic pump.	0.1% SDS0.1% Triton X100 for 24–72 h0.4% Sodium deoxycholine for 24–72 h ± 90 U/mL benzonase for 2 h.	Not reported.	0.2 and 0.4 mL/min for 12, 24, 48 or 72 h	PBSPBS/PenStrep for 1 h.	Time.	1–2 days	Acellular kidney provided regionalized factors that are highly instructive, resulting in organized kidney structures within the acellular kidney.	Sambi et al. (2017)

Porcine	Vessels were cannulated and attached to a peristaltic pump, followed by overnight perfusion of ddH_2_O.	1% of the detergent Triton X-100 and 0.1% ammonium hydroxide in ddH_2_O.	Not reported.	Not reported.	10–60 mL/h for 24 h or until translucent. Perfused with ddH_2_O prior to sterilization (gamma irradiation).	The removal of the cellular components is observable with the transparency/white color of the decellularized bioscaffolds.	2 days.	Preserved vascular network.	Baptista et al. (2009)

Porcine	Perfused with 10 USP units/mLSodium heparin in 1× PBS for 15 min at 0.75 L/h.	0.5% SDS in 1× PBS, 0.25% SDS in 1× PBS, or 1% TritonX-100/0.1% Ammonium Hydroxide in 1× PBS were perfused through the kidneys for a total of 36 h.	Not reported.	0.75 L/h.	1× PBS for 48 h;500 mL of DNase solution (0.0025 w/w% DNase in 1× PBS at neutral pH) overnight;Rinse with 1× PBS for 1 h;10.0 kGy gamma irradiation to sterilize the decellularized scaffolds.	Time.	6–7 days.	Advantages for the use of 0.5% SDS in the decellularization of kidneys of a clinically relevant size.	Sullivan et al. (2012)

Porcine	Rinsed with heparinized PBS.	1% (v/v) SDS in dH_2_O.	Not reported.	Approximately 100 mL/min.	PBS solution was perfused for 24 h.	White appearance.	2–3 days.	Preservation of major architecture and vasculature.	Park and Woo (2012)

Porcine	PBS	1% Triton X-100 or 1% SDS.	Not reported.	Not reported.	dH_2_O.	Transparency.	Not reported.	Verified that 1% Triton X-100 is a more suitable decellularizing agent than SDS regarding structural, biochemical integrity and biocompatibility of the scaffold.	Chae et al. (2014)

Porcine	Perfused with heparinized 1× PBS solution.	0.5% SDS.	80 mmHg.	Not reported.	dH_2_O.	Time.	2–3 days.	Freeze porcine kidneys prior to decellularization to prevent spoilage by bacterial growth and after decellularization to prevent proteins from denaturing. The decellularized organs can then be preserved for months without cryoprotectants and thawed just prior to recellularization.	Poornejad et al. (2015)

Porcine	Perfusion of heparinized PBS.	dH_2_O;1% SDS;1% Triton X-100.	Not reported.	10 mL/min.	PBS.	Translucency.	3–4 days.	Scaffolds maintain their basic components and show intact vasculature system.	Guan et al. (2015a)

Porcine	Saline solution.	1.0% Triton X-100; PBS; 0.25% or 0.75% SDS;	80 mmHg.	1 L/h.	PBS;DNase;1% antibiotics/antimycotics;Sterilized with 1% MIN-NCARE^®^ (4.5% PAA and 22.0% hydrogen peroxide), or by irradiation with 12–16 kGy over 24–30 h.	Time.	4 days.	Maintenance of distinct vascular and collecting system compartments.	Willenberg et al. (2015)

Porcine	Heparinized (PBS) solution was perfused into the kidneys through the catheterized artery to prevent thrombosis. The harvested kidneys were then preserved at −20°C.	Solutions of 0.1 N NaOH (pH 11.8–12), 1% (w/v) PAA (pH 2.6), 3% (v/v) Triton X-100 (pH 7.2), 1% (w/v) SDS (pH 8.1), and 0.05% Trypsin/ethylenediaminetetraacetic acid (EDTA).	Bench top shaker (70–80 r/min)	Bench top shaker (70–80 r/min)	Deionized (DI) water wash.	Time	24 h.	The NaOH solution induced the most efficient cell removal and resulted in the highest amount of cell viability and proliferation after recellularization, although it also produced the most significant damage to collagenous fiber networks. The SDS solution led to less severe damage to the ECM structure but it resulted in lower metabolic activity and less proliferation. PAA and Triton X-100 resulted in minimum disruption of ECMs and the most preserved glycosaminoglycans (GAGs) and FGF. However, these last two agents were not as efficient in removing cellular materials as NaOH and SDS, especially PAA, which left more than 80% of cellular material within the ECM.	Poornejad et al. (2016b)

Porcine	The kidneys were removed with special care to ensure that a sufficient length of renal artery was preserved. Heparinized PBS solution was perfused into the kidneys through a catheter to prevent thrombosis. The harvested kidneys were then preserved at −20°C until decellularization. After thawing overnight at 4°C, fat was stripped from the renal capsule, excess arterial tissue was excised, and the kidneys were cannulated *via* the renal artery with white nylon tubing.	Hypertonic solution (0.5 M NaCl in H_2_O) was pumped into the kidneys for 30 min.0.5% w/w SDS solution for 30 min, followed by DI water (hypotonic solution) for 30 min.	80 mmHg	Began at 10 mL/min and was incrementally increased every 30 min by 1.5 mL/min to approximately 40–50 mL/min.	DI water wash.	White kidney	2 days.	Preservation the microstructure while still removing 99% of the DNA.	Poornejad et al. (2016a)

Porcine Yorkshire	Washed twice with PBS.	1% Triton X-100 or 1% SDS containing 100 U/mL penicillin and 100 μg/mL streptomycin. Samples were decellularized at 4°C in a shaking incubator (200 rpm) Sullivan et al. (2012)	Not reported.	Not reported.	DNase (30 μg/mL) in PBS for 1 h;Decellularized kidney scaffolds were cryo-embedded in optimum cutting temperature compound.	Transparency.	10–14 days.	Identified 1% Triton X-100 as a more suitable decellularizing agent for porcine renal ECM scaffolds prior to kidney regeneration.	Choi et al. (2015)

Porcine (Bama miniature)	Perfused with perfusate NaCl 8.3 g/L, KCl 0.5 g/L, HEPES 2.4 g/L, and EGTA (0.95 g/L). Rinsed twice with PBS and stored at −20°C until use.	dH_2_O;1% SDS; Triton X-100; PAA; NaDOC.	Not reported.	15 mL/min.	PBS.	Time.	1–2 days.	Clearance of cellular components and xenoantigens, including DNA and protein and preservation of the ECM composition.	Wang et al. (2014)

Rat	Renal arteries were cannulated immediately after euthanization of the animal and perfused with PBS with vasodilator (10 mL of 10 lg/mL sodium nitroprusside in PBS, Sigma/UK, followed by 20 mL at 1 μg/mL) until a uniform blanching was observed, after which each kidney was perfused with 30 mL PBS without vasodilator.	SDS at differing concentrations and durations (1.0, 0.125, 0.25, and 0.5%).	Not reported.	10 mL/min	PBS for 1 h	Time.	4 hat 24 h	Improved preservation of both structural and functional components of the whole kidney ECM bioscaffold.	He et al. (2016)

Rat	Heparinized PBS for 15 min.	1% SDS for 12 h, ddH_2_O for 12 min, 1% Triton X-100 for 30 min, PBS for 48 h, and antibiotic-containing PBS.	100 cmH_2_O.	10–40 mL/min.	ddH_2_O.	Time.	3 days.	No cell residue was found in the scaffolds under microscope.	Liu et al. (2015a)

Rat	Anesthetized rats were systemically anti-coagulated with heparin, and cannulas were inserted in the renal artery and ureter. The kidney was arterially perfused *in situ* using a saline solution containing a vasodilator (nitroprusside) to remove the blood. The organ was observed to confirm uniform blanching indicative of even distribution of perfusate and was harvested.	NaDOC as the ionic detergent:Triton X-100 at 0.5, 3, 6, and 10% solutions; ddH_2_O; DNase;4% NaDOC;Use of SDS:3% Triton X-100, DNase, repeat 3% Triton X-100, and then the 4% SDS.0.05% sodium azide.	Approximately 100 mmHg (perfusion system was gravity based to).	Not reported.	ddH_2_O.	Time.	5 days.	Both of the detergent-based perfusion protocols successfully produced acellular kidneys that were nearly transparent, yet retained the web-like appearance of the basement membrane.	Ross et al. (2009)

Rat	Systemic heparin anticoagulation;Saline solution containing a vasodilator (nitroprusside);Continuous gravity-based perfusion.	Multiple sequential solutions that included Triton X-100 and SDS detergents, DNase, and deionized water rinses.	Approximately 100 mmHg.	Not reported.	ddH_2_O.	Not reported.	Over 5 days.	New evidence for matrix-to-cell signaling in acellular whole organ scaffolds that induces differentiation of pluripotent precursor cells to endothelial lineage.	Ross et al. (2012)

Rat	Not reported.	1% Triton X-100;1% Triton X-100 + 0,1% SDS;1% Triton X-100 + Trypsin 0.02%-EGTA 0.05%.	Not reported.	1 mL/min;5 mL/min	Not reported.	Not reported.	2 days.	Only Triton/SDS and Trypsin-EGTA/Triton Protocols successfully removed cells while preserving the architecture and components of the ECM in rat kidneys. Triton/SDS Protocol retained a higher amount of FGF compared to the other protocols. Triton/SDS Protocol efficiently removed cells while maintaining scaffold structure and retaining growth factors that may be critical for transplant and reseeding of the scaffold with renal and endothelial progenitor cells.	Caralt et al. (2013)

Rat	Systemic heparinization;Perfusion of heparinized PBS at 30 mmHg arterial pressure for 15 min.	12 h of 1% SDS in ddH_2_O, 15 min of ddH_2_O, and 30 min of 1% Triton-X-100 in ddH_2_O.	30 mmHg constant pressure.	Not reported.	Washed the kidney scaffolds with PBS containing 10,000 U/mL penicillin G, 10 mg/mL streptomycin, and 25 μg/mL amphotericin-B at 1.5 mL/min constant arterial perfusion for 96 h.	Not reported.	4 days.	Yield acellular scaffolds with vascular, cortical, and medullary architecture, collecting system and ureters.	Song et al. (2013)

Rat	Kidney was perfused using a saline solution (NaCl 0.9%) containing a vasodilator (nitroprusside, 10^−4^ M).	1% SDS in PBS for 17 h at a flow rate of 0.4 mL/min;	Physiological range (from 62 ± 16–107 ± 23mmHg).	0.4 mL/min.	ddH_2_O.	Not reported.	17 h.	Rat kidneys can be efficiently decellularized to produce renal ECM scaffolds in a relatively short time and rapid recellularization of vascular structures and glomeruli.	Bonandrini et al. (2014)

Rat	50 U/mL heparin in 0.01 M PBS, pH 7.4 for 30 min.	0.1% TritonX-100 for 3 h, ddH_2_O for 30 min, 0.8% (v/v) SDS for 3 h, and ddH_2_O containing 100 U/mL penicillin and 100 mg/mL streptomycin for 24 h.	Not reported.	8 mL/min.	Kidney scaffolds were kept in 50 mL of ddH_2_O containing the penicillin and streptomycin at 4°C for less than 7 days.	Not reported.	8 days.	Decellularized kidney scaffolds could be used to promote renal recovery in the treatment of chronic kidney disease.	Yu et al. (2014)

Rat	Kidneys were harvested without previously administering anticoagulation medication to the animals. Kidneys were frozen at −80 C in PBS.	ddH2O for 10 min.1st:SDS concentrations of 0.25, 0.5, 0.66, and 1% combined with a perfusion time of 0.5, 1, 2, and 4 h.2nd:concentration of SDS was always 0.66% and the perfusion time was 1 h. After the first 30 min of perfusion with SDS, the kidneys were washed for 10 min with dH_2_O and then the organs were perfused for another 30 min with the SDS solution.	100 mmHg.	Not reported.	ddH_2_O for 1 h. Decellularized organs were perfused for 1 h under sterile conditions with recirculated, sterilely dH_2_O containing 200 U/mL of penicillin and 200 mg/mL of streptomycin.	Not reported.	5 h.	Novel standardized, time-efficient and reproducible protocol for the decellularization of solid tissues to derive a ready-to-use biomatrix within only 5 h.	Burgkart et al. (2014)

Rat	0.01 M PBS, pH 7.4, for 15 min.	0.5% SDS.	Not reported.	Approximately 2 mL/min.	PBS.	Time.	1–2 days.	Successfully produced renal scaffolds by decellularizing rat kidneys with 0.5% SDS, while still preserving the ECM 3D architecture, an intact vascular tree and biochemical components.	Guan et al. (2015b)

Rat	dH_2_O.	Protocol 1:1% Triton X-100;Protocol 2:1% Triton X-100; 0,1% SDS;Protocol 3:0.02% Trypsin-0.05% EGTA; 1% Triton X-100.	Not reported.	Not reported.	dH_2_O.	Time.	1–2 days.	Triton and Triton/SDS preserved renal microarchitecture and retained matrix-bound basic FGF and vascular endothelial growth factor. Trypsin caused structural deterioration and growth factor loss. Triton/SDS-decellularized scaffolds maintained 3 h of leak-free blood flow in a rodent transplantation model and supported repopulation with human iPSC-derived endothelial cells and tubular epithelial cells *ex vivo*.	Caralt et al. (2015)

Rat	Cold PBS was perfused until all blood was cleared. All organs were stored in PBS at–20°C. All organs were gradually thawed at room temperature.	1% Triton X-100, 1% Triton X-100/0.1% SDS and 0.02% Trypsin-0.05% EGTA/1% Triton X-100 according to Caralt et al. (2015)	Not reported according to Caralt et al. (2015)	Not reported according to Caralt et al. (2015)	Stored scaffolds in PBS at 4°C for a maximum of 2 weeks prior to use.	Translucency.	7 days.	Demonstrated non-invasive monitoring capabilities for tracking dynamic changes within scaffolds as the native cellular component is removed during decellularization and model human cells are introduced into the scaffold during recellularization and proliferate in maintenance culture.	Uzarski et al. (2015)

Rhesusmonkeys (*Macacamulata*)	Washed with PBS.	1% SDS.	Not reported.	Not reported.	Washed with PBS and stored in 1% (v/v) penicillin–streptomycin in PBS at 4°C until use (2 months maximum).	Transparency.	10–14 days.	Decellularized scaffolds have an intrinsic spatial ability to influence hESC differentiation by physically shaping cells into tissue-appropriate structures and phenotypes, and additional approaches may be needed to ensure consistent recellularization throughout the matrix.	Nakayama et al. (2013)

Rhesusmonkeys (*Macacamulata*)	All tissues were placed in DMEM. Kidney sections were washed twice with PBS.	1% (v/v) SDS or 1% (v/v) Triton X-100 diluted in dH_2_O at either 48°C or 37°C. Decellularization solution was changed 8 h after the initial tissue harvest and then every 48 h until the tissues were transparent.	Not reported.	Not reported.	Washed with PBS.	Transparency.	7–10 days.	SDS was the most effective for decellularization of kidney sections. Triton X-100 was unable to completely decellularize the tissues and caused greater disruption of the basement membrane and connective tissue ECM.	Nakayama et al. (2010)

Rhesusmonkeys (*Macacamulata*)	All tissues were placed in DMEM upon collection with processing conducted at the time of tissue harvest. Kidney sections were washed twice with PBS.	1% (v/v) SDS diluted in dH_2_O at 4°C. The solution was changed 8 h after initial tissue harvest and then every 48 h until the tissue was transparent.	Not reported.	Not reported.	washed with PBS;10% (v/v) penicillin/streptomycin (Gibco, Invitrogen) in PBS at 4°C until use.	Transparency.	7–10 days.	Removal of cellular components while preserving the structural and functional properties of the native ECM.	Nakayama et al. (2011)

*ECM, extracellular matrix; ddH_2_O, double-distilled water; PBS, phosphate-buffered saline; SDS, sodium dodecyl sulfate; NaDOC, sodium deoxycholate; DNase, deoxyribonuclease; DMEM, Dulbecco’s modified Eagle’s medium; EGTA, ethylene glycol tetraacetic acid; FGF, fibroblast growth factor; hESCs, human embryonic stem cells; dH_2_O, distilled water; NaCl, sodium chloride; KCl, potassium chloride; HEPES, 4-(2-hydroxyethyl)-1-piperazineethanesulfonic acid; PAA, peracetic acid; iPSC, induced pluripotent stem cell*.

## Elements That Influence the Decellularization Process

The decellularization processes require sensitive methods due to the fragility of the organs and their internal structural complexity. Therefore, it is necessary to develop techniques for decellularization and residual DNA removal simultaneously to preserve the integrity of the ECM. There are several factors that influence the process of decellularization, such as cell density, body weight, lipid content, the organ thickness, and the intrinsic properties of the substances employed to remove the cells (Ross et al., 2009). Furthermore, it is necessary to consider the mechanical forces involved in the process, such as the pressure of perfusion, flow rate, and possibility of the use of retrograde infusion of fluids (Lin et al., 2016). Another point to be considered is that the methods used in the organs of small animals (e.g., mice) have to be adapted to larger animals, such as monkeys and pigs, which resemble humans, at least regarding the size of the organ. In this sense, some studies have already demonstrated good results with the decellularization of monkey’s and pig’s kidneys, with preservation of the ECM scaffolds (Badylak et al., 2009; Orlando et al., 2011b, 2012; Sullivan et al., 2012).

Regarding the types and concentrations of detergents employed in the decellularization process, we considered that each detergent exhibits a distinct pattern of action depending on the tissue involved (Baptista et al., 2009). There are two detergents widely used currently—Triton X-100 and SDS—at concentrations ranging from 0.1 to 5%, perfused alone or in combination (Baptista et al., 2011; Barakat et al., 2012; Sullivan et al., 2012; Nakayama et al., 2013). Recently, He et al. (2016) compared the success of rat kidney decellularization through SDS perfusion in different combinations of time of perfusion (4 and 8 h) and concentration of SDS (0.125, 0.25, 0.5, and 1.0%), with the best result obtained with 0.125% SDS perfused through 4 h. In this condition, histological and immunohistochemical evaluation were not different from other conditions, but growth factor maintenance was more efficient. Another recent approach to decrease SDS exposure time and improve the decellularization process of porcine kidneys employed a combination of freezing/thawing, low concentrations of SDS, incremental increases in flow rate under constant pressure, and applying osmotic shock to the cellular membranes. These procedures preserved the microstructure of the scaffold while still removed 99% of the DNA, enhanced cell–ECM interactions, and allowed the seeded cells to grown more rapidly when compared to kidneys decellularized only with SDS perfusion for longer period (Poornejad et al., 2016a). This same group compared the effect of five different decellularization agents on porcine renal tissue (0.1 N NaOH, 1% PAA, 3% Triton X-100, 1% SDS, and 0.05% trypsin/EDTA). While the NaOH solution was the most efficient on cell removal and cell viability after recellularization, it damaged the ECM components, besides it also produced lots of caustic waste. This could be avoided with the use of HCl to neutralize NaOH. In turn, SDS led to less severe damage to ECM structure, but the cell viability and proliferation after recellularization were not as efficient as the NaOH solution. Triton X-100 and PAA preserved ECM structure and growth factors, although these reagents were not as effective as the SDS and NaOH on cell removal (Poornejad et al., 2016b). In summary, the NaOH solution was considered the best choice if the intention is implantation of cells and rapid biodegradation of the scaffold. Nevertheless, adopting a multistep decellularization protocol using ionic and non-ionic detergent exposure would probably result in better preservation of a relatively intact structure.

## Decellularization Assessment

**Figure 1:**
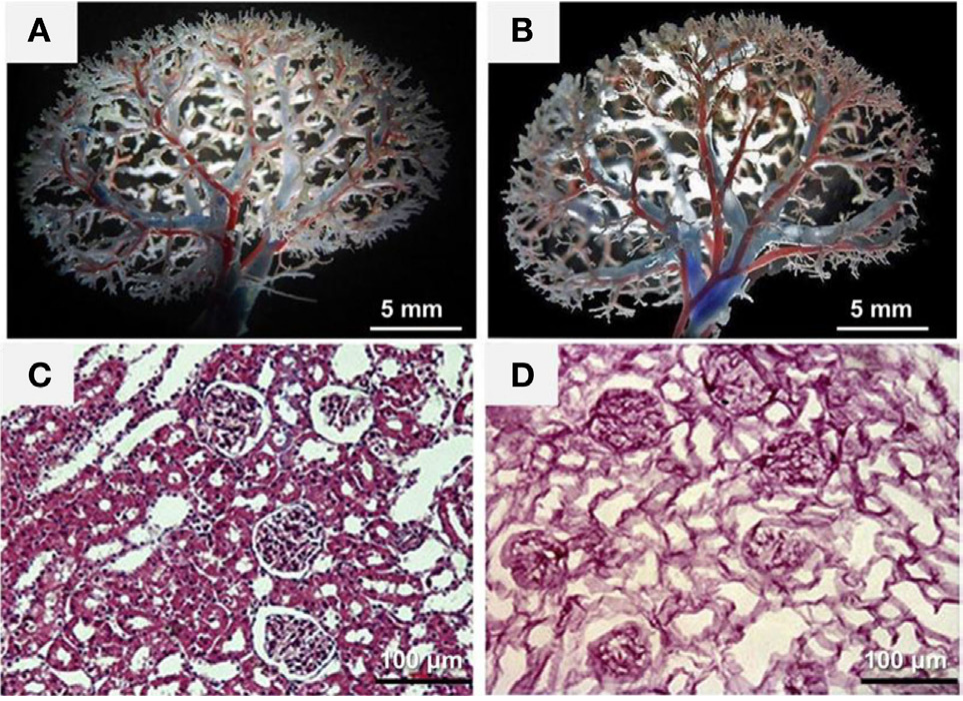
**Characterization of the decellularized kidney scaffolds**. **(A,B)** Vascular corrosion casting showed a normal vascular tree of the decellularized kidney scaffold **(B)** compared with that of the intact kidney **(A)**. **(C,D)** H&E staining showed the existence of blue-stained nuclei in the intact kidney **(C)** but not in the decellularized kidney scaffold **(D)**. Reprinted and modified with permission from Yu et al. (2014).

Maintaining the architecture and composition of the ECM is the largest challenge to the success of the decellularization process. With respect to the ECM composition, although many groups have demonstrated the retention of collagen, laminin, elastin, and fibronectin after decellularization (Caralt et al., 2015), the reduction or depletion of ECM proteins and growth factors has also been reported (Singh et al., 2010; Akhyari et al., 2011; Petersen et al., 2012; Wallis et al., 2012; Caralt et al., 2015). The retention of the main components of the ECM, such as collagen and laminin, provides the preservation of the ultrastructure of the scaffold, which can facilitate repopulation by providing the necessary spatial orientation (Scarritt et al., 2015). To evaluate the ECM structure maintenance, a system for histological classification is usually applied to verify whether the fundamental characteristics of renal scaffolds were preserved after decellularization. This system may include assessment of the ECM structure of tubules, glomeruli, and vessels, as well as the level of removal of cells (Figures 1C,D). Transmission electron microscopy has also been used to demonstrate whether the structure of the glomerular basement membrane was preserved after decellularization (Goh et al., 2013; Orlando et al., 2013). As a further evaluation of the retention of ECM components, many researchers have employed the use of traditional engineering techniques such as atomic force microscopy and uni- or bi-axial mechanical tests to evaluate the biophysical properties of the decellularized organ. In many cases, decellularization affects the rigidity of the matrix due to the removal of cells and damage to ECM components (Ott et al., 2008, 2010; Nakayama et al., 2010; Price et al., 2010; Wainwright et al., 2010; Daly et al., 2012; Petersen et al., 2012; Goh et al., 2013). The evaluation of the growth factor retention rate may also be a pattern that indicates the biofunctionality of the ECM (Caralt et al., 2015). Recent studies have shown that preservation of the ECM induces pluripotent cells cultured in the decellularized scaffolds to differentiate into tissue-specific cells (Ross et al., 2009; Cortiella et al., 2010). This cell differentiation linked to ECM intrinsic properties allows the engineering of complex tissues. Moreover, the composition of the ECM is dynamic and constantly changing in response to the metabolic activity of living cells, which modulate their own niche (Badylak et al., 2011). Withal the *in silico* predication of the genes that encode the ECM components and cell proteins that interact with, or modify the ECM, resulted in a database called “*the matrisome*” (Naba et al., 2012; Randles et al., 2017). Proteomic analysis by the use of mass spectrometry analysis focused on the matrisome, allow a complete identification and quantification of the ECM components, and already represent powerful resources for researchers (Gessel et al., 2015; Hill et al., 2015; Calle et al., 2016).

In addition to the parenchymal structures, maintaining the acellular scaffold microvasculature is critical for subsequent recolonization. The perfusion of dyes followed by angiographic analyses with micro computed tomography or vascular corrosion are useful strategies to evaluate the structure of the vascular tree after decellularization and have been performed in kidneys (Figures 1A,B) (Ott et al., 2008; Shupe et al., 2010; Uygun et al., 2010; Baptista et al., 2011; Barakat et al., 2012; Sullivan et al., 2012; Bonvillain et al., 2013; Caralt et al., 2013; Goh et al., 2013; Mirmalek-Sani et al., 2013; Yu et al., 2014). The complete removal of the DNA from the ECM donor is important to prevent a possible inflammatory response in its implementation into the host after recellularization (Badylak et al., 2009; Gilbert et al., 2009). Currently, the techniques for evaluating the presence of DNA in the ECM include spectrophotometric analyses (Rosario et al., 2008; Narayanan et al., 2009), histological stains (Bolland et al., 2007; Rosario et al., 2008; Gilbert et al., 2009), and immunohistochemical methods (Woods and Gratzer, 2005). The quantification of beta-actin gene (one of the main non-muscular cell components) is another reliable alternative method to assess the presence of DNA in the ECM (Kabsch et al., 1990). Furthermore, the beta-actin gene has been widely used as a positive control in polymerase chain reactions (PCRs) (Bellis et al., 2003) and is also configured as a pattern in gene tests due to its constitution and ubiquitous expression (Nygard et al., 2007).

The decellularization techniques developed until the present moment have not been able to remove 100% of the native cellular components without damage to the ECM structure and composition. The residual DNA content may cause cytocompatibility problems *in vitro* and adverse events in hosts during the reintroduction of cells *in vivo* (Nagata et al., 2010; Zhang et al., 2010). The threshold concentration of residual cellular components within the ECM, which is sufficient to cause the host response, depends on the (Lai et al., 2013) source of the ECM (United States Renal Data System, 2015), immune response of the receiver, and (Jha et al., 2013) environment in which the ECM will be installed. In this regard, the minimum criteria that reflect a satisfactory decellularization process involve displaying less than 50 ng of double-stranded DNA per mg of ECM (dry weight) with fragments lower than 200 base pairs and the absence of visible nuclear material on optical microscopy stained with 4′,6-diamidino-2-phenylindole (DAPI) or hematoxylin (Figure 1) (Woods and Gratzer, 2005; Crapo et al., 2011). Thus far, there is no consensus regarding the use of a specific detergent because this can degrade collagen, even in similar tissues, thus decreasing the mechanical strength of the organ (Cartmell and Dunn, 2000; Woods and Gratzer, 2005).

## Scaffold Sterilization

Prior to use *in vitro* or *in vivo*, the ECM scaffold must be sterilized to remove all endotoxins and possible viral or bacterial DNA. The solvents or acidic solutions may be used for this process (Hodde and Hiles, 2002; Gorschewsky et al., 2005). However, these solutions can damage the ECM and affect the adhesion of cells during repopulation (Sun and Leung, 2008). The use of gamma irradiation or ethylene oxide is also reported, but these approaches also alter the ultrastructure of the ECM/scaffold, with the loss of collagen fibers and GAGs, decrease in cell proliferation and alterations in porosity and swelling properties (Poornejad et al., 2016c). Furthermore, exposure to this type of process can activate immune responses from the receptor (Qiu et al., 2009). One alternative for the sterilization of the kidney scaffold without damage to the ECM is the infusion of antibiotic and antifungal solutions, such as sodium azide or a combination of penicillin, streptomycin, and amphotericin, generally diluted in sterile phosphate-buffered saline (PBS). Perfusion with sterile filtered 70% ethanol is also used before perfusion with antibiotics (Nakayama et al., 2010, 2011; Xu et al., 2014; Poornejad et al., 2015). The kidney scaffold is usually perfused with the antimicrobial solution over a period of time to ensure that no microorganisms remain in the scaffold (Nakayama et al., 2010, 2011; Song et al., 2013; Bonandrini et al., 2014). Recently, Poornejad et al., 2016c showed that a 0.2% PAA in 1 M NaCl solution was the best method for decontamination of porcine decellularized ECM over other sterilization solutions such as 70% ethanol and 0.2% PAA in 4% ethanol or gamma-irradiation.

## Cell Sources for Kidney Recellularization

The kidney is a complex organ that contains more than 26 types of cells derived from ureteric bud and the metanephrogenic mesenchyme (MM) (Al-Awqati and Oliver, 2002; Nishinakamura, 2008; Gouon-Evans, 2014). The renal corpuscle is composed of endothelial cells, mesangial cells, visceral epithelial cells, also known as podocytes, and parietal epithelial cells, besides the cells that comprise the juxtaglomerular apparatus, such as peripolar cells, juxtaglomerular granular cells, extraglomerular mesangial cells, and cells from the macula densa (Barajas, 1979). The renal proximal tubular cells are epithelial cells that can be distinguished in different cell types, according to the proximal tubular segment in which they are located. In rats, the proximal tubular segments are divided into S1, S2, and S3 segments, and the cells that comprise these segments have been well described by Maunsbach (1966). At the loop of Henle, the epithelial cells are also distinguished by their ultrastructural characteristics and their location within the different regions that form the medulla (Pannabecker, 2012). There are also cells that form the distal tubules, composed of morphologically distinct segments—the thick ascending limb of the loop of Henle, the macula densa, the distal convoluted tubule, and connecting tubules. Each of these segments presents different epithelial cells, with different functions related to their ultrastructural characteristics. Finally, the cells that comprise the collecting duct are the principal cells, type A and type B intercalated cells, and cells that form the inner medullary collecting duct, in addition to the different types of interstitial cells (Little et al., 2007).

In view of this complexity and variety of cells that form the kidneys, different strategies have been used to recellularize this organ. An immortalized human renal cortical-tubular epithelial (RCTE) cell line seeded through the renal artery within a bioreactor has also formed tubular structures and was not found within arterioles, suggesting that cells could translocate into the parenchyma or peri-tubular space to rest on the basement membrane (Caralt et al., 2015).

Another approach to repopulate the kidney scaffold is based on the contribution to urine production from various epithelial cell phenotypes presented in different niches along the nephron (Song et al., 2013). The infusion of suspended human umbilical venous endothelial cells (HUVECs) *via* the renal artery and a combination of rat neonatal kidney cells (NKCs) *via* the ureter is a promising strategy. These cells can be maintained in culture media supplemented with *in vivo* maturation signals such as glucocorticoids and catecholamines to accelerate *in vitro* nephrogenesis and the maturation of NKCs in acellular kidney matrices. In this sense, repopulation of the renal scaffold with epithelial and endothelial cells with the preservation of glomerular, tubular, and vascular architecture was observed through histologic evaluation after approximately 4 days in culture (Song et al., 2013). HUVECs lined vascular channels throughout the entire scaffold cross section, and the spatial organization of the regenerated epithelium and endothelium resembled the native nephron, which could provide the anatomic basis for renal function, including the processes of water and solute filtration, secretion, and reabsorption (Song et al., 2013).

Primary adult renal cells isolated from kidney cortical tissue by a process of digestion have also been applied to kidney recellularization (Abolbashari et al., 2016). Prior to seeding into the kidney scaffold, the isolated cells were maintained in primary culture, and the expression of aquaporin 1, aquaporin 2, aquaporin 4, ezrin, and podocin was analyzed by immunostaining to identify the cell phenotypes. The majority of the cells were positive for aquaporin1 and ezrin (a protein localized at the brush border membrane of proximal tubules that cross-links plasma membrane proteins with the actin cytoskeleton), indicative of proximal tubular cells, while distal tubular cells and collecting duct cells, expressing aquaporin 2 and aquaporin 4, respectively, represented a small percentage of the total cell population, with very few cells expressing podocin. After the identification of the phenotypes that confirmed the presence of cells from different renal segments, the cells were seeded into the scaffold. Some functional determinations such as electrolyte and protein adsorption, hydrolase activity, and erythropoietin (EPO) production were accessed to evaluate the functional capacity of the engineered kidneys, and the results were encouraging. However, they did not focus on the full characterization of the cultured cells, and cells with vascular phenotypes were not used, leading to the need for future tests to be performed (Abolbashari et al., 2016).

Embryonic stem (ES) cells have been used for organ recellularization with good results (Ross et al., 2009, 2012; Bonandrini et al., 2014). ES cells are especially interesting because of their pluripotency and ability to grow indefinitely. These cells have the potential to form any embryonic organ *in vivo*, and their capacity to differentiate into any of the adult renal cell types makes these cells great candidates for kidney recellularization (Keller, 2005). Pluripotent ES cells are usually maintained in culture medium in the presence of leukemia inhibitory factor, which reduces spontaneous cell differentiation, until the moment of the recellularization (Garreta et al., 2014). Infusion of the cells in the kidney scaffold through both the renal artery and ureter shows a pattern of distribution of the injected ES cells into tubular and vascular structures and their associated glomeruli, with multiplication of the cells (Song et al., 2013). Furthermore, evidence of embryonic cell differentiation toward epithelial and endothelial phenotypes was obtained by immunohistochemistry and fluorescence microscopy (Ross et al., 2009, 2012; Guan et al., 2015b). When the ES cells are infused only by the renal artery, the cells remain largely in the vascular network, with few cells housed on the tubular segments (Bonandrini et al., 2014).

Despite the good results obtained in applying ES cells for organ recellularization, ethical questions and the teratogenic potential of ES may limit the use of these cells for organ repopulation (Nowacki et al., 2014). One possible alternative is the use of autologous adult stem cells that do not require direct ethical questions, do not lead to immunologic responses, and can be obtained in minimally invasive processes. Bone marrow mesenchymal stem cells (BM-MSCs) have frequently been used for kidney repair and regeneration (Burgkart et al., 2014), but stem cells from other sources such as adipose tissue or amniotic fluid also exhibit promising applications (Hass et al., 2011; Liu et al., 2015b). Induced pluripotent stem cells (iPSCs) can be generated directly from adult somatic cells *via* the transduction of reprogramming factors and closely resemble ESCs been also suitable for kidney regeneration (Rogers, 2011; Hendry and Little, 2012; Usui et al., 2012). Some studies have achieved highly efficient differentiation of human iPSCs into intermediate mesoderm cells with potential to generate embryonic renal progenitors and adult renal cell types (Mae et al., 2013), as well as iPSC differentiation into podocytes with cytoplasmic contractile response to angiotensin II (AII) and functional ability to albumin intake (Song et al., 2012). The reconstitution of kidney structures *in vitro*, including efficiently vascularized glomeruli with podocytes, as well as renal proximal and distal tubules with clear lumen have already been generated from iPSCs differentiated to MM (Taguchi et al., 2014). A schematic review detailing the differentiation of MM from iPSCs was published from this same group of researchers (Taguchi and Nishinakamura, 2014). Furthermore, iPSC-derived endothelial cells injected through the renal artery of decellularized kidney were able to outlining branching vasculature and individual glomeruli (Caralt et al., 2015). It can be considered that patient-decellularized kidneys reseeded with patient-derived iPSCs differentiated to the renal precursors and vascular progenitors may represent the most promisor recellularization option to the reconstruction of whole kidneys that can hereafter be used for autologous transplantation.

## Whole-Organ Recellularization

Kidney recellularization is usually made by the anterograde infusion of cells through the vasculature or retrograde infusion through the ureter or both (Ross, 2009; Ross et al., 2009, 2012; Song et al., 2013; Bonandrini et al., 2014). The cells can be manually injected (Ross et al., 2009, 2012), or they may be subject to infusion using a syringe pump (Bonandrini et al., 2014) or peristaltic pump (Song et al., 2013; Caralt et al., 2015), which allows for control of the flow rate of infusion. These latter options, coupled to a bioreactor, seem to be more adequate because they ensure the constant infusion of cells without apparent damage to the scaffold. In addition, maintaining a constant or pulsatile perfusion of cell culture medium allows the viability, nutrition, proliferation, migration, and differentiation of the recent seeded cells (Scarritt et al., 2015). Another alternative to seeding the renal scaffolds is the delivery of the cells into the cortical region of renal scaffolds using a needle. The distance between the injection sites and depth of injection has to be optimized to ensure that the whole organ is covered, with cells homogeneously injected into the cortex site (Abolbashari et al., 2016).

**Figure 2:**
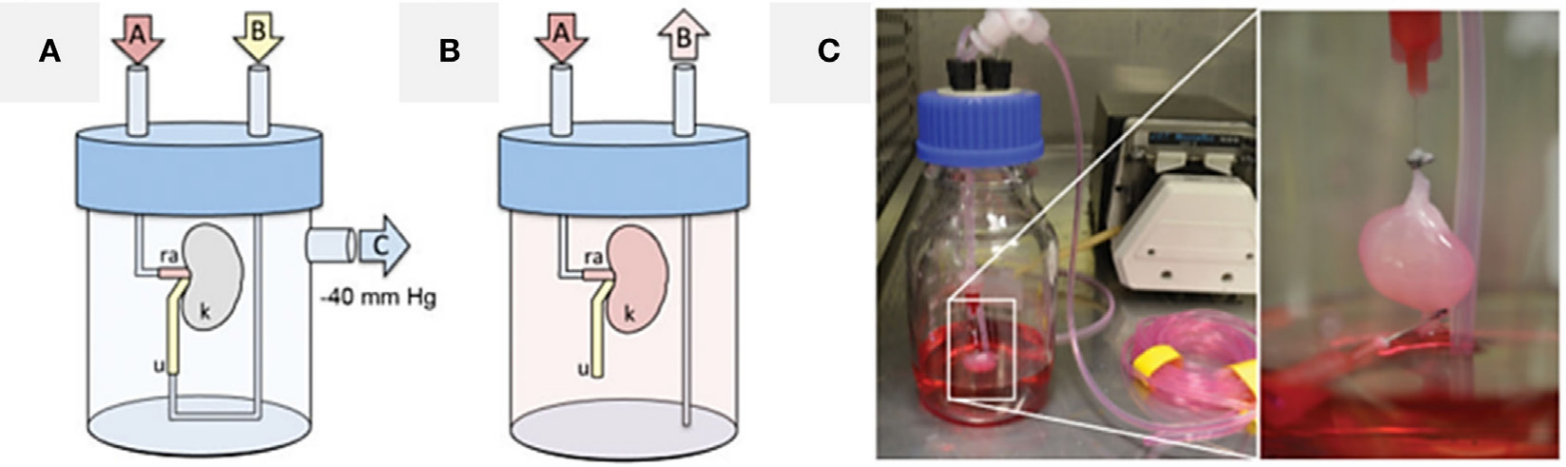
**Cell seeding and whole-organ culture of decellularized rat kidneys**. **(A)** Schematic of a cell seeding apparatus enabling endothelial cell seeding *via* port A attached to the renal artery (ra) and epithelial cell seeding *via* port B attached to the ureter (u), while negative pressure in the organ chamber is applied to port C, thereby generating a transrenal pressure gradient (left). **(B)** Schematic of a whole-organ culture in a bioreactor enabling tissue perfusion *via* port A attached to the renal artery (ra) and drainage to a reservoir *via* port B (u, ureter; k, kidney). **(C)** Cell seeding of decellularized rat kidneys in whole-organ culture. Reprinted and modified with permission from Song et al. (2013).

The first attempt to recellularize a whole kidney used the incubation of the reseeded organ with static media. This procedure allowed less reproducible growth, significant apoptosis occurred, and this approach did not consistently maintain viability beyond approximately 4 days. Better results were observed with transverse sectioning of the pre-seeded organ and culture of the slices in a multiwell dish, but this approach cannot be extrapolated to rebuild the organ for transplantation (Ross et al., 2009). Still, a peristaltic pump coupled to appropriate tubing could deliver a physiologically normal pressure profile, in a temperature-controlled incubator supplied by regulated medical-grade sterile gases. In this case, there was sufficient cell viability and proliferation with migration into glomeruli and other small vessels, extending from the afferent arterioles and glomerular tufts to the efferent arterioles and peritubular capillary networks (Ross et al., 2009, 2012). Other attempts to seed cells by applying pressure to the collecting system failed to reach the glomerulus, encouraging the development of a bioreactor for the perfusion of cells through the renal artery and retrograde through the ureter, with a vacuum pressure of 40 cmH_2_O applied to the system (Figure 2). This approach ensured cell seeding with a transrenal gradient and culminating in cell dispersion throughout the entire kidney parenchyma (Song et al., 2013). Thereafter, other studies also used perfusion-based bioreactors for renal repopulation with considerable success (Bonandrini et al., 2014; Caralt et al., 2015; Abolbashari et al., 2016). A table with a summary of some methods and cell sources already used for kidney repopulation can be seen in the review published by Scarritt et al. (2015).

## Characterization of the Reseeded Kidney Structure

The characterization of the reseeded kidney with respect to the ECM structure, cell adhesion, proliferation, migration, and differentiation, as well as the regeneration of a new kidney, that resembles the native structure is usually made by histological evaluation. Monitoring the distribution of the cells allows for observation not only of cell proliferation and migration through the scaffold but also the integrity of the vascular basement membranes based on the presence or absence of cells at the Bowman’s space (Ross et al., 2009, 2012; Song et al., 2013; Caralt et al., 2015). Morphometric analysis is also described for repopulated kidney characterization, such as the analysis of the average glomerular diameter, Bowman’s space, and glomerular capillary lumen (Song et al., 2013). Elastin staining gives the notion of vessel and artery integrity (Caralt et al., 2015). Transmission and scanning electron microscopy are also performed to evaluate the final structure of the rebuilt kidney, showing glomerular capillaries with engrafted podocytes and the formation of foot processes (pedicels) (Song et al., 2013; Caralt et al., 2015).

**Figure 3:**
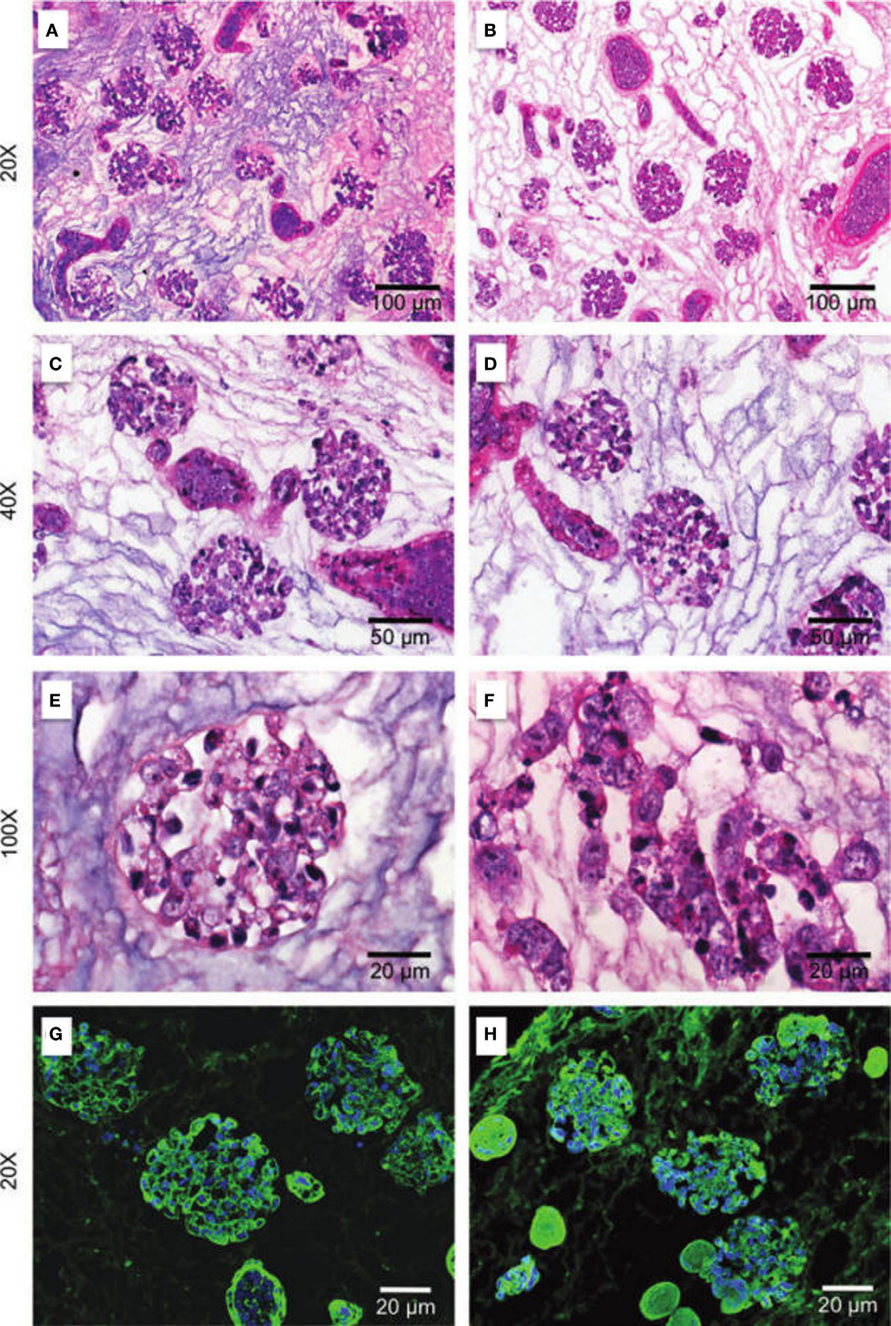
**Repopulation of kidney scaffolds with mES cells**. Hematoxylin and eosin staining at 24 h **(A,C,E)** and 72 h **(B,D,F)** and immunostaining for WGA agglutinin and DAPI at 24 h **(G)** and 72 h **(H)** in kidney scaffolds seeded with mES cells show a homogeneous distribution of cells into glomerular and vascular structures, peritubular capillaries, and tubules. mES, murine embryonic stem cells. Reprinted and modified with permission from Bonandrini et al. (2014).

Histological staining with H&E performed for light microscopy allows the analysis of cell distribution, as well as the maintenance of ECM structure (Figure 3). Observations relative to the morphology of the engrafted cells can indicate their cytology features and assessment of the distribution, migration, and grouping tendencies, as well as cell–ECM interactions indicate the success of the chosen method (Ross et al., 2009; Song et al., 2013; Bonandrini et al., 2014; Caralt et al., 2015; Abolbashari et al., 2016). The use of GFP-stained cells for recellularization is also a strategic tool for the characterization of the distribution of the cells along the kidney scaffold by monitoring GFP expression by fluorescence microscopy (Ross et al., 2009, 2012). Other stains are also used to access cell distribution, such as the periodic acid-Schiff (PAS) stain, in addition to fluorescein wheat germ agglutinin (WGA) and DAPI staining, which documents cellular nuclei in the scaffold (Figure 3) (Ross et al., 2009; Bonandrini et al., 2014; Caralt et al., 2015).

In addition to the histological stains, the evaluations of the expression of cell differentiation and proliferation markers by immunostaining or RT-PCR are also tools for the characterization of the rebuilt kidney. In this regard, there is a range of antibodies available for review of different cell types, resulting in repopulation of kidney scaffolds. Some of the proteins and genes accessed for evaluation of cell differentiation are Oct-4, a marker of embryonic stemness, NCAM, a marker of mesoderm precursors, and the endothelial cell markers Tie-2 and CD3 (Bonandrini et al., 2014). Pan-cytokeratin immunostaining and staining of Pax-2 and Ksp-cadherin, a cell adhesion protein normally expressed in distal nephron tubular cells at later developmental stages, may also be useful in evaluating stem cell differentiation into epithelial tubular cells (Ross et al., 2009). Lectin-specific (BsLB4) staining for murine endothelial cells and the presence of VEGFR2 are indicators of the differentiation of ES cells into endothelial cells in the scaffold’s glomeruli and blood vessels (Ross et al., 2009, 2012). Markers such as Wilms tumor WT-1, podocalyxin like-2, glial cell-derived neurotrophic factor (GDNF), LMX1B, nephrin, and synaptopodinlike-2 are other indications of mature renal cells (Petrosyan et al., 2015). For a more accurate observation of the specific cells differentiated upon recellularization, analysis of the expression of podocin indicates a glomerular epithelial phenotype (Abolbashari et al., 2016), while the polarity of the cells with expression of (Na^+^)/(K^+^)-ATPase represents a proximal tubular phenotype (Song et al., 2013). E-cadherin expression can be used as a marker of the distal tubular phenotype, and the expression of β1-integrin indicates glomerular epithelial site-specific cell adhesion to ECM domains. Furthermore, as mentioned previously in the “Cell sources for kidney recellularization” section, the evaluation of the different aquaporins can also indicate the level of differentiation of the tubular cells in the different segments of the kidney (Song et al., 2013; Abolbashari et al., 2016). The expression of aquaporin1 together with ezrin indicates the differentiation of cells into proximal tubular cells, while distal tubular cells and collecting duct cells express aquaporin2 and aquaporin4, respectively (Hatano et al., 2013; Abolbashari et al., 2016).

Cell proliferation and viability are other important parameters to ensure that recellularization is successful. Cell viability is usually assessed by immunostaining of BAX, an apoptotic activator, or cleaved caspase-3, a critical executioner of apoptosis (Ross et al., 2009; Bonandrini et al., 2014). Another approach to assess cell apoptosis is TUNEL staining (Abolbashari et al., 2016). The analysis of cell proliferation, in turn, is made by staining with Ki-67, a nuclear protein necessary for cellular proliferation (Bullwinkel et al., 2006; Ross et al., 2009; Abolbashari et al., 2016) and proliferating cell nuclear antigen (PCNA), a co-factor for DNA polymerase δ activity in eukaryotic cells, that acts as a scaffold for the recruitment of proteins involved in DNA repair, chromatin remodeling, and DNA replication (Moldovan et al., 2007; Bonandrini et al., 2014; Abolbashari et al., 2016). The PCNA/BAX ratio is also used to confirm the success of the cell seeding over time (Petrosyan et al., 2015).

Functional analysis of the rebuilt kidney is also performed. The electrolyte reabsorption can be monitored by sodium uptake assays, with slices from the recellularized kidney cortex incubated with fluorescent sodium dyes and imaged using a fluorescent microscope. For this purpose, ouabain may be used to inhibit (Na^+^)/(K^+^)-ATPase on the cells, while normal kidney may be used as a positive control (Abolbashari et al., 2016). Albumin uptake can also be measured using the same methods adapted to sodium uptake, indicating the functionality of proximal tubular cells (Birn and Christensen, 2006). Amino acid transport is another important function performed by renal cells. This activity can be evaluated by accessing the activity of hydrolases, such as leucine aminopeptidase (LAP) and gamma glutamyltranspeptidase (GGT), which play significant roles in amino acid transfer (Meister, 1974; Abolbashari et al., 2016).

Secretome analysis is also an excellent approach to assess the functionality of the recellularized organ (Petrosyan et al., 2015). By analyzing the ability of the cells to secrete various proteins, it is possible to estimate the rate of differentiation of the seeded cells and their metabolic activities. Analysis of metalloproteinases and their inhibitors (MMPs and TIMPs) indicates the induction of cells to produce matrix modulators and their potential to repair and remodel the ECM. The secretion of growth factors (VEGF, TGF, PDGF) is related to different tissue processes, including matrix remodeling, immunomodulation, and angiogenesis. Cytokine (interleukins and GRO) and chemokine (MCP-1, RANTES, Fractalkine) secretion are indicative of multiple processes, including inflammation, angiogenesis, wound healing, and immunomodulation (Peloso et al., 2015; Petrosyan et al., 2015). Another pattern that can be observed to characterize renal function is the EPO production by renal cortical interstitial cells through real-time measurement of secreted EPO into the cell culture medium during bioreactor culture (Ullrich, 1979; Abolbashari et al., 2016).

Finally, transmission electron microscopy images may complement the analyses of cell functions, through the evaluation of the intensity of expression in the endoplasmic reticulum and Golgi apparatus, as well as the presence of secretory granules and vacuoles within the cells, which together are indicative of cellular activity and secretion, demonstrating the ability of cells to synthesize key molecules involved in tissue and organ homeostasis (Petrosyan et al., 2015). Alternatively, the development of non-invasive techniques for monitoring cell behavior such as surface scanning or multiphoton microscopy would be very important to improve the evaluation of the success of the recellularization process (Scarritt et al., 2015). Actually, some currently non-invasive techniques used in regenerative medicine may be used in tissue engineering, such as the resazurin perfusion assay, which allows the repetitive and rapid estimation of viable cell numbers during long-term *ex vivo* culture without destruction of the organ (Ren et al., 2015). Another non-invasive approach used in ECM scaffold remodeling evaluation is the use of a ^19^F-MRI (magnetic resonance imaging) contrast agent in ^1^H-MRI scans, which can adequately monitor the distribution of transplanted cells while allowing an evaluation of the formation of new tissue (Bible et al., 2012).

## Future Challenges

**Figure 4:**
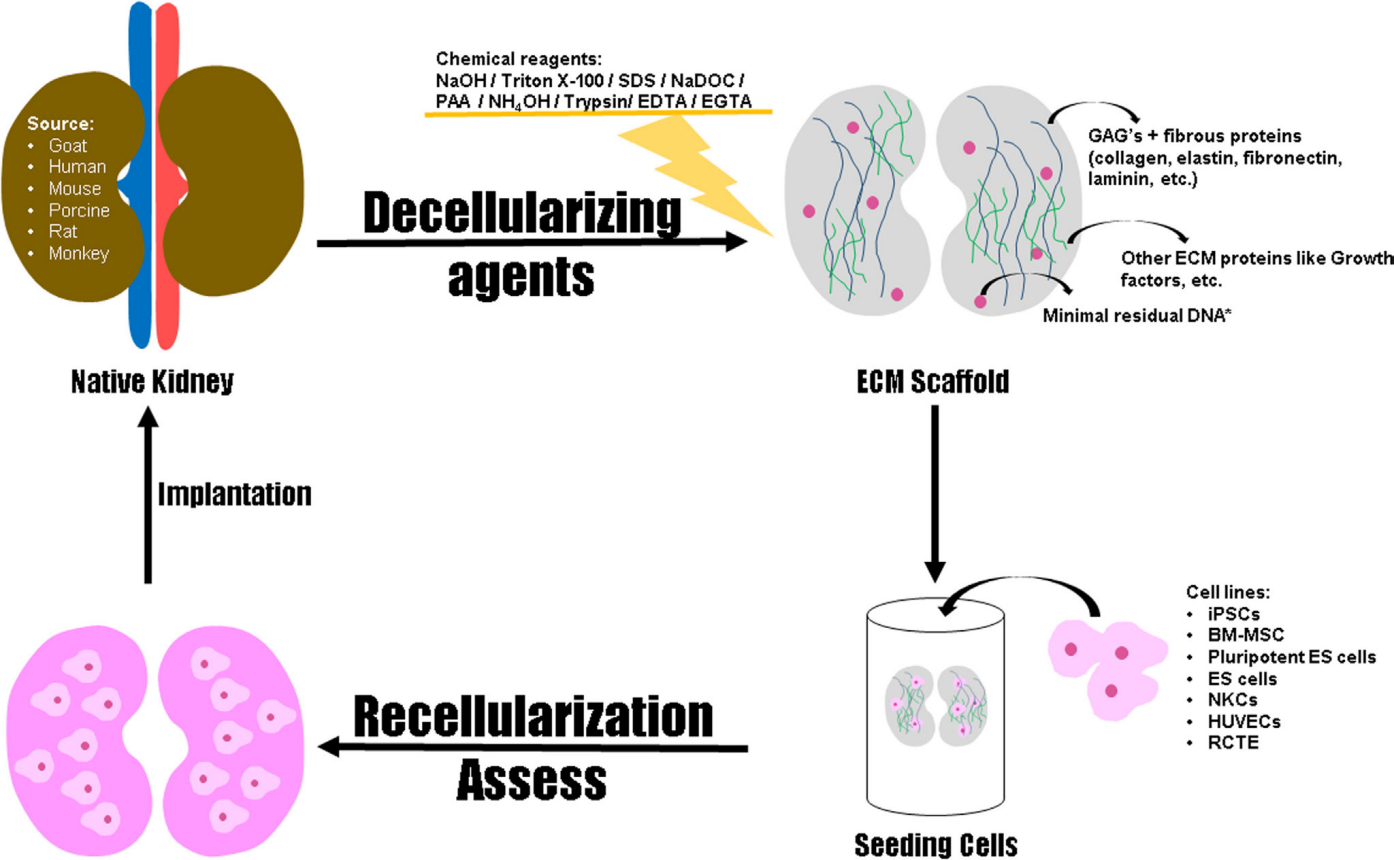
**Schematic representation for the development of functional organ by decellularization and recellularization**. Native kidneys provided from goat, human, mouse, porcine, rat, and monkey can be decellularized through biological, physical, or chemical methods aiming to obtain a scaffold with preserved structural integrity, retention of ECM proteins and other elements (i.e. growth factors). Regarding the cells types used for repopulation of the scaffold, studies using immortalized human renal cortical-tubular epithelial (RCTE) cell line, human umbilical venous endothelial cells (HUVECs), neonatal kidney cells (NKCs), Embryonic stem (ES) cells, pluripotent ES cells, Bone marrow mesenchymal stem cells (BM-MSCs) and induced pluripotent stem cells (iPSCs) have been driven. The number of adhered cells, the level of cell differentiation, and the functionality of the bioengineered organ must be evaluated for posterior implantation through allotransplantation or xenotransplantation. *Scaffold displaying less than 50 ng of double-stranded DNA per mg of ECM (dry weight) with fragments lower than 200 base pairs.

We have reviewed one of the promising technologies in regenerative therapies for entire organ recovery. Solid organ regeneration based on perfusion-decellularized native ECM scaffolds has enormous potential in patients with organ failure. The use of biologic scaffolds can represent a potential alternative in the field of tissue engineering and regenerative medicine since the employed methods for decellularization can achieve success in the removal of cells without damaging the ECM. It is now necessary to improve these methods to extrapolate them for large-scale experiments. Figure 4 represents the development of functional organ by decellularization and recellularization, indicating the decellularizing agents, the ECM components to be evaluated post decellularization and the cell sources for recellularization of the organ.

Many of the present efforts aimed at progressive strategies for cell expansion and differentiation might deliver original solutions applicable to organ engineering in the foreseeable future. Considering the several types of cells that have been used in different approaches for kidney recellularization research, choosing the ideal cell source will determine the effectiveness of the new building organ when *in vivo* transplantation occurs. Until now, ES cells have seemed to be the most efficient cell source for kidney recellularization. On the other hand, there are some ethical issues that make ES cells an unsuitable option for human organ recellularization and transplantation. With these considerations in mind, there is a need for more studies regarding iPSCs or adult stem cells, such as bone marrow-derived stem cells or adipocyte-derived stem cells, which present no ethical issues but have not yet been used in transplantation post-recellularization. iPSCs represent an ideal alternative to the ES cells, presenting the same ability of ES to efficiently expand in culture, as well as their capacity to differentiate into multiple cell types, with the advantage of having no ethical problems.

Although several methods have been used to characterize the success of kidney regeneration after decellularization, these processes are often directed toward histological characterization; thus, there is a lack of information regarding the functionality of the new kidney. The newly formed organ should mimic the same biological functions of the native organ without impairing its functionality; and this is what is expected for the next decade (Peloso et al., 2016). The *in vitro* capacity of regenerated kidneys to filter a standardized perfusate, clear metabolites, reabsorb electrolytes and glucose, and generate concentrated urine was assessed by Song et al. (2013), and the results indicate a partial recovered function of endothelial cells, podocytes, and tubular epithelial cells (Song et al., 2013). Their results could be related to incomplete seeding and the immature stage of seeded neonatal epithelial cells (FALK, 1955; Baum, 2009). Despite the functional immaturity observed through *in vitro* analysis, *in vivo* transplantation showed that regenerated kidney constructs provided urine production and clearance of metabolites, without the occurrence of bleeding or graft thrombosis (Song et al., 2013). However, this method has presently achieved only short-term functionality *in vivo* and only in rodent models. Until now, there has been no report of a mature complete regenerated kidney successfully transplanted into a donor with recovery of its functions, an observation that is a challenge in the field of kidney bioengineering at present. Recently, Caralt and colleagues investigated the ability of a decellularized kidney scaffold to hold sutures and support systemic arterial blood flow by implanting it into a rat recipient in an orthotopic position. Although there was an absence of clotting or major bleeding and the kidney scaffolds remained intact *in situ* for the duration of the study, these results cannot be extrapolated to a real transplantation, considering the absence of cells in the scaffolds (Caralt, 2015; Caralt et al., 2015).

In view of all of the advances observed in kidney bioengineering concerning organ decellularization and repopulation, there is a huge perspective regarding the confirmation of recovery of kidney function after transplantation. The expectation of researchers for the next 10 years is that the studies with decellularized and recellularized kidneys are in the preclinical phase, representing a high potential for translation compared to other tissue engineering approaches to kidney repair, such as renal organoids or 3D bioprinting (Peloso et al., 2016). Nonetheless, the adaptation of the methods described to date for use in human kidney regeneration and transplantation would change the current perspective related to the treatment of kidney diseases in the near future.

## Author Contributions

AD and GS: each author has made a valuable contribution to the preparation of this manuscript such as writing, literature review, and table layout. The authors AD and GS contributed equally to this work. BN: conception of this work, drafting the work, and revising it critically for all content.

## Conflict of Interest Statement

The authors declare that the research was conducted in the absence of any commercial or financial relationships that could be construed as a potential conflict of interest. The reviewer, MH, and handling editor declared their shared affiliation, and the handling editor states that the process nevertheless met the standards of a fair and objective review.
